# Mind the gap! Risk factors for poor continuity of care of TB patients discharged from a hospital in the Western Cape, South Africa

**DOI:** 10.1371/journal.pone.0190258

**Published:** 2018-01-25

**Authors:** Lilian Dudley, Fidele Mukinda, Robin Dyers, Frederick Marais, Dagmar Sissolak

**Affiliations:** 1 Division of Health Systems and Public Health, Faculty of Medicine and Health Sciences, Stellenbosch University, Cape Town, South Africa; 2 Department of Global Health, Harvard Chan School of Public Health, Boston, Massachusetts, United States of America; 3 Department of Health, Western Cape Provincial Government, Cape Town, South Africa; 4 Department of Infection Control, Medical disaster control and Environmental health control, Department of Public Health, Berlin Central, Germany; Agencia de Salut Publica de Barcelona, SPAIN

## Abstract

**Background:**

TB patients discharged from hospitals in South Africa experience poor continuity of care, failing to continue TB treatment at other levels of care. Factors contributing to poor continuity of TB care are insufficiently described to inform interventions.

**Objective:**

To describe continuity of care and risk factors in TB patients discharged from a referral hospital in the Western Cape, South Africa.

**Design:**

This retrospective observational study used routine information to describe continuity of care and risk factors in TB patients discharged from hospital.

**Results:**

788 hospitalized TB patients were identified in 6 months. Their median age was 32 years, 400 (51%) were male, and 653 (83%) were urban. A bacteriological TB test was performed for 74%, 25% were tested for HIV in hospital, and 32% of all TB patients had documented evidence of HIV co-infection. Few (13%) were notified for TB; 375 (48%) received TB medication; 284 (36%) continued TB treatment after discharge; 91 (24%) had a successful TB treatment outcome, and 166 (21%) died. Better continuity of care was associated with adults, urban residence, bacteriological TB tests in hospital and TB medication on discharge. Fragmented hospital TB data systems did not provide continuity with primary health care information systems.

**Conclusions:**

Discharged TB patients experienced poor continuity of care, with children, rural patients, those not tested for TB in hospital or discharged without TB medication at greatest risk. Suboptimal quality of hospital TB care and a fragmented hospital information system without linkages to other levels underpinned poor continuity of care.

## Introduction

Tuberculosis (TB) remains a leading cause of preventable morbidity and mortality globally[1, 2]. South Africa, with a TB incidence of 834/100 000 is ranked by WHO as one of the 30 high TB burden countries [1]. The country adopted the DOTS TB control strategy in 1995, delivering ambulatory TB treatment at primary health care (PHC) facilities. However, the HIV epidemic in the 1990’s drove a rising TB incidence rate [3], resulting in an HIV prevalence of 57% in incident TB cases in 2016 [4, 1]. The HIV epidemic and growing TB drug resistance with 3.5% of new TB cases having multiple drug resistant (MDR) TB) [1] has increased hospital TB admissions in South Africa since the 1990’s [5].

Hospitalized TB patients in many countries receive poor quality of care and are lost to follow up due to weak linkages between hospitals and other levels of care [6–9]. South African studies found that less than 50% of TB patients discharged from public hospitals continued treatment at PHC facilities [10, 11]. Socio-demographic, epidemiologic and health system differences across provinces [12], and a lack of analysis of risk factors for continuity of care of patients discharged from hospitals limited generalisation from these earlier studies. This study was therefore undertaken in a 1300 bed Central Academic Hospital (CAH) receiving up to 50% of tertiary referrals from a catchment population of 5.4 million (2009) in the Western Cape. In the pre-HIV era this Province had the highest TB prevalence in South Africa, largely due to poor socioeconomic conditions and failed public health responses before and during apartheid [13].

The Provincial TB incidence of 906/100 000 at the time of this study remained one of the highest in the country [12].

Given the increasing role of hospitals in responding to the TB epidemic, we aimed to describe risk factors for continuity of TB treatment on discharge from hospital to inform local interventions to improve continuity of care for TB patients.

## Study population and methods

This observational study was a retrospective review of records of CAH TB patients over a six month period (November 2008 to April 2009) and their outcomes post discharge (November 2008 to April 2010). Patient demographic and treatment data were extracted from routine hospital information systems, including a clinical administrative information system (Clinicom), the hospital’s National Health Laboratory Services (NHLS) information and a national NHLS data warehouse, the hospital pharmacy database, notifications and death certificates in a hospital administration file. The collated data were validated against a 10% random sample of patient folders. This revealed unreliable ICD classification in Clinicom [14], and patients with a TB ICD code in Clinicom which was not verified by NHLS, pharmacy, notification or death certificate data were excluded from the study.

Outcome data on continuity of care, defined as whether discharged TB patients continued treatment at public sector PHC service or specialised TB hospitals in the Western Cape, were collected from the ETR.net (Electronic TB Register) and EDR.web (Electronic Drug Resistant TB Register). A one year follow up period allowed for completion of National TB Programme (NTP) treatment regimens for new and retreatment TB patients and ETR.net reporting timeframes. Hospital information and TB registers did not share patient identifiers, and patient records were matched using probability data linkage on name, gender, age, area of residence, and date of treatment [15]. The ETR.net and EDR.web data were extracted by two data managers and compared for completeness. Discharged TB patients not found through electronic data linkage were sought by manually searching the ETR.net and paper-based quarterly TB registers at district offices. Data were collated, cleaned and quality checked in Excel, and analysed in STATA 13 [16]. A biostatistician provided statistical support.

Patient demographic variables included gender, age, race, employment status, and area of residence. Disease variables included HIV status, type of TB infection, and TB drug sensitivity. Hospital clinical management included length of stay (LOS), number of admissions during the study period, TB and HIV diagnostic tests, treatment for TB and HIV, and TB notification.

The main continuity of care outcome variable was patient registration for TB treatment at another public health facility in the Western Cape. Secondary outcomes included NTP treatment outcome in the TB registers, and mortality as captured in all data sources.

All data except age were treated as categorical variables. Age was treated as a continuous variable and categorised into patients < 15 years old (children) and those > = 15 years (adults). We report univariate analyses of explanatory and outcome variables; bivariate analyses using Chi2 tests for associations between explanatory and outcome variables; and multiple logistic regression analysis.

Ethical approval was obtained from the Faculty of Medicine and Health Sciences, University of Stellenbosch (Ref: N09/05/149), and IRB exemption for data analysis from Harvard TH Chan School of Public Health (IRB17-0109). Permission for the study was also obtained from the CAH, the Health Departments of the Provincial Government of the Western Cape and the City of Cape Town.

## Results

A total of 874 TB patients were identified from hospital information systems for the six month period based on an ICD code for TB and/or a record of TB diagnosis, treatment, notification or death. Of these, 86 (9.8%) lacked sufficient proof of TB and were excluded from the study.

**Fig 1:**
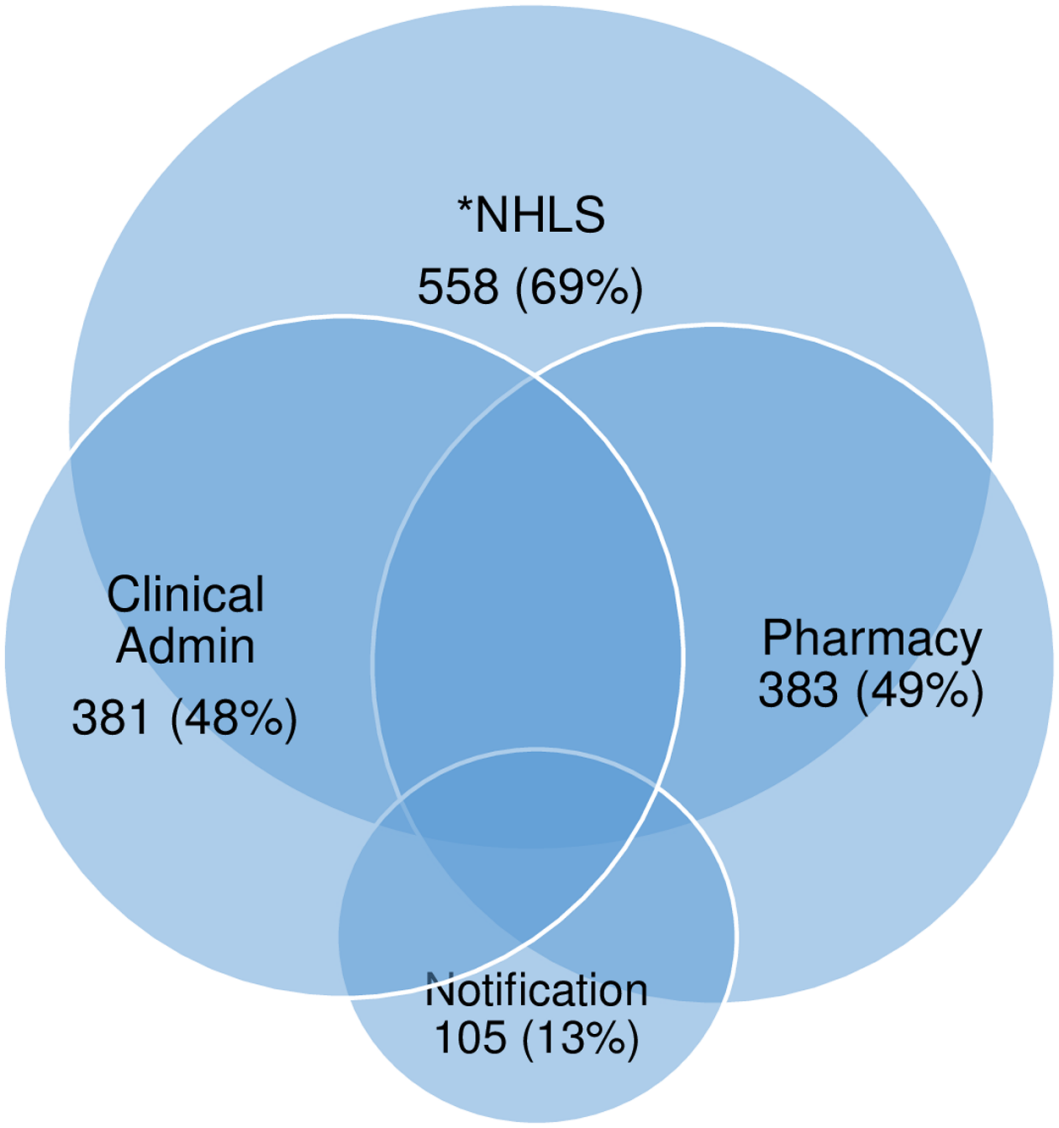
Percentage of hospital TB patients (N = 788) identified from different hospital databases. NHLS = National Health Laboratory Services.

Of the 788 included TB patients, 381(48.2%) were in Clinicom, 548(69.4%) in the NHLS database, 383(48.5%) in the hospital pharmaceutical information system, and 105(13.3%) from hospital TB notifications. Although the data sets overlapped, none included all TB patients. Fig 1.

The mean and median age of hospital TB patients was 30.6 and 32 years (IQR 21–42), with 150 (19%) under 15 years of age, 400 (50.8%) male, and 429 (54.4%) had race categorised as black and 342 (43.4%) as mixed race. Most (653, 82.9%) lived in the City of Cape Town, with 135 (17.1%) from rural areas. Table 1. Males represented 63% of rural TB patients, differing significantly from urban TB patients which included more females (52%) (Chi2 p<0.001). Of the 497 adults (77.7%) with employment information, 286 (57.5%) were unemployed and 47 (9.5%) received a social grant.

**Table 1:** Description of TB patients treated in hospital in a 6 month period, outcomes after discharge, and associations between continuity of care and explanatory variables.

Variables	Hospital TB patients (N = 788)		Main Outcome		
			Continued TB care (N = 284)	Did not continue TB care (N = 504)	Chi^2^ P value
	n	%			
**Age** (years)					
<15	150	*19*	28	122	< 0.001
> = 15	638	*81*	256	382	
**Gender**					0.537
Male	400	*50*.*8*	140	260	
Female	388	*49*.*2*	144	244	
**Race**					0.646
Black	429	*54*.*4*	160	269	
Mixed race	342	*43*.*4*	120	222	
Other	17	*8*.*5*	4	13	
**Residence**					<0.001
Urban	653	*82*.*9*	255	398	
Rural	135	*17*.*1*	29	106	
**Single Admission**	431*	67.3	-	-	
**LOS**† (median, IQR)	11*	5.0–23	-	-	
**TB Bacteriology**			112	103	<0.001*
Smear &/or culture	586	*74*.*4*			
Drug sensitivity	358	*45*.*4*			
Mono -R	24	*6*.*7*^‡^			
MDR	20	*5*.*9*^‡^			
XDR	7	*2*^‡^			
**TB Medicine Provided**	375	*47*.*6*	131	244	0.537
**TB Notification**	105	*13*.*3*	40	65	0.638
**HIV co-infection**	253	*32*	106	147	0.024
**Arrived PHC**	284	*36*			
Completed TB Rx	125	*15*.*9*			
Cured	66	*8*.*3*			
Died	36	*4*.*6*			
Defaulted	26	*3*.*3*			
Transferred out	11	*1*.*4*			
Treatment failure	7	*0*.*9*			
**Total Died**	166	*21*.*1*	58	108	0.740

* n = 640.

^†^ Length of Stay.

^‡^of patients tested.

A total of 586 (74.4%) patients had TB bacteriology tests, 550 in hospital and an additional 36 in the NHLS warehouse reflecting tests done in hospital or PHC services. Of these 513 (65.1%) had a positive TB smear and/or culture result, with 19 with positive smears alone. Urban patients (75%) had more hospital TB bacteriology tests than rural patients (57%) (p<0.001). Of the 358 (45.4%) patients tested for TB drug sensitivity, 307 (86.0%) were sensitive to all TB drugs, 24 (6.7%) were mono resistant to Rifampicin or INH, 20 (5.9%) had MDR, and 7 (2.0%) had XDR TB. Table 1.

Only 196 (24.9%) TB patients had an HIV diagnostic test in hospital, of whom 69 (35.2%) were positive. A further 141 (17.9%) had a CD4 count or viral load test in hospital. HIV co-infection was documented for 253 (32%) TB patients based on hospital HIV related tests or ART treatment. This differed significantly by race, with 169 (39.4%) black and 80 (23%) mixed race patients HIV co-infected (p<0.001). Females had non-statistically significant higher HIV infection rates (35.5%) than males (28.5%) (p = 0.09). Rural TB patients had significantly lower HIV infection rates (18.5%) than urban patients (34.9%) (p = 0.001).

TB medication was issued to 375 (47.6%) of all TB patients. However only 224 (40.7%) of the 550 hospital bacteriologically diagnosed TB patients had a hospital TB medicine prescription. Of the 159 patients issued TB medication without a hospital bacteriology test, many were children (30%) or from rural areas (28.3%). Of the 105 (13.3%) notified TB patients, 64 were female (16.5%) and 41 male (10.3%) (p = 0.01). More patients with hospital bacteriological TB tests were notified (78, 13.8%) than patients without such tests (25, 11.6%), (Fisher’s Exact p = 0.035).

Of 640 (81.2%) TB patients with information on number of admissions, 431 (67.3%) had one, 129 (20.2%) two and 80 (12.5%) ranged from 3 to 15 admissions during the six months. Their median LOS was 11 days, ranging from 1 to 143 days (IQR 5.0 to 23.0).

### Outcomes

Only 284 (36%) of the 788 TB patients continued TB treatment in the Western Cape. Of these, 207 (72.9%) were recorded in the ETR.net or EDR.net, and 77 (27.1%) in the facility TB register, but not in the electronic registers. Three patients (1%) were discharged to the Eastern Cape without records of outcomes.

**Fig 2:**
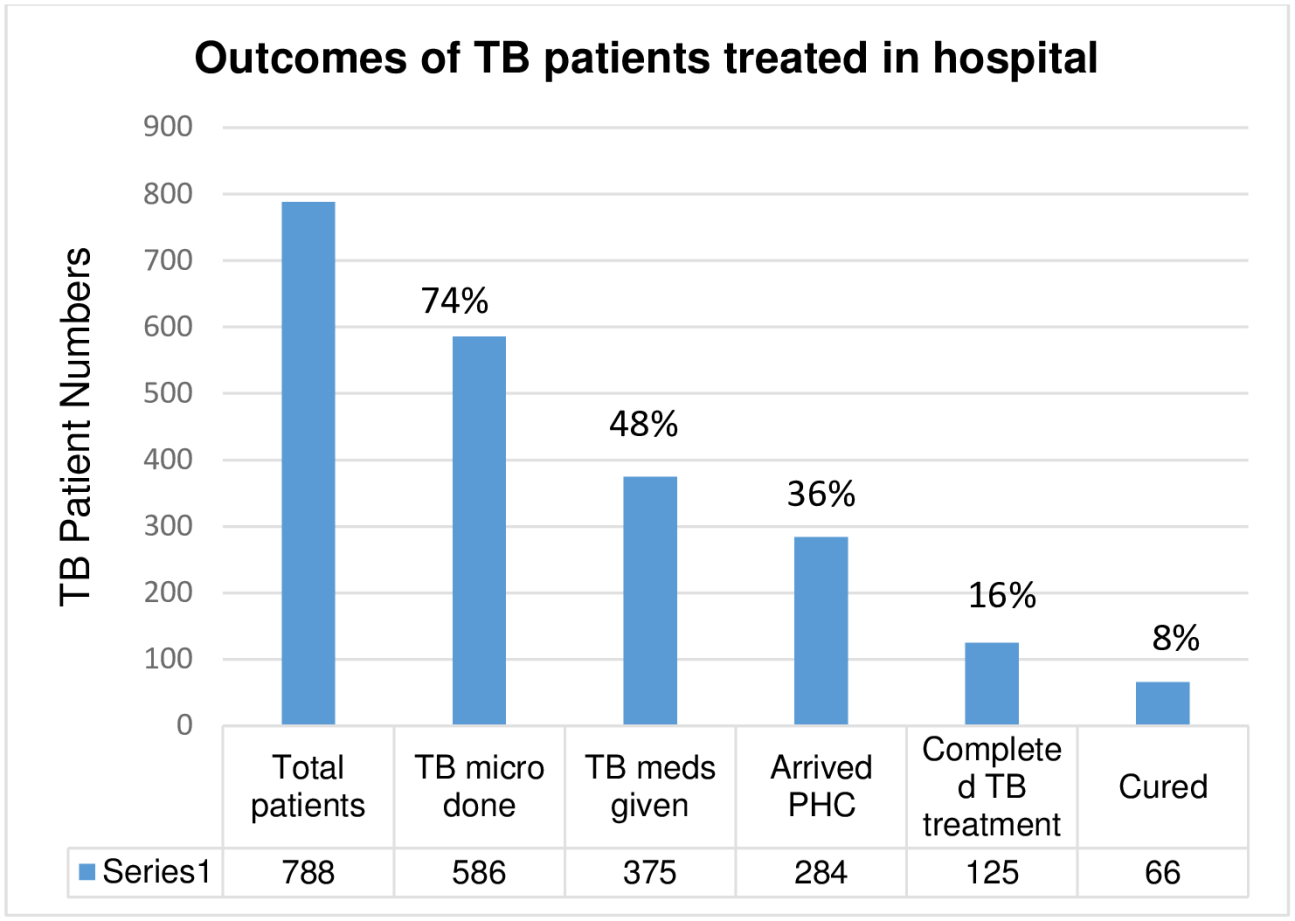
Outcomes of TB patients (N = 788) treated in a hospital in the Western Cape, South Africa.

A total of 125 (15.9%) patients completed TB treatment and an additional 66 (8.3%) were cured, totalling 191 (24.2%) with successful treatment outcomes. Another 36 (4.6%) patients died, 26 (3.3%) defaulted, 11(1.4%) transferred out, and 7(0.9%) were treatment failures based on the TB registers. Fig 2 illustrates the proportions of hospital TB patients with the respective outcomes.

More adults (256; 40%) than children (28; 18.7%) (p<0.001); urban patients (255; 39.0%) compared to rural (29; 21.5%) (p <0.001); more known HIV co-infected patients (106; 40.1%) (p = 0.024); and patients who had a TB bacteriology test in hospital (p<0.001) continued TB care at other facilities. Continuity of TB care was not associated with gender, race, or notification status. Table 1.

A total of 166 deaths (21.1%) were captured from all data sources. Mortality was higher in HIV co-infected patients (27.7%) than in patients without evidence of HIV infection (16.4%) (p = 0.006). Adults (134, 21.5%) were more likely to die than children (16, 9.6%) (p = 0.001), and notified patients (11.4%) less likely than those not notified (22%) (p = 0.009). There were no significant associations between mortality, gender, race, area of residence, and TB bacteriology tests.

An analysis excluding 91 (11.5%) patients who had died in hospital or within 14 days of discharge, found similar demographic, treatment and outcomes to the overall patient group, with only 262 (37.6%) continuing treatment at other levels of care. Improved continuity in this group was associated with adults (p <0.001), urban residence (p<0.001), and TB bacteriology in hospital (p<0.001). Risk factors for death after discharge included adult age (p = 0.042) and HIV co-infection (p = 0.005). Table 2.

**Table 2:** Outcomes of hospital TB patients who were discharged and alive 14 days post discharge.

Variables	Hospital TB patients discharged and alive 14 days post discharge (N = 697)		Continued Care (N = 262)	Chi^2^ P value	Died (N = 77)	Chi^2^ P value
	n	%	n		n	
**Age** (years)						
<15	143	20.52	27	<0.001	9	0.042
> = 15	554	79.48	235		68	
**Gender**				0.590		0.667
Male	355	50.93	132		41	
Female	342	49.07	130		36	
**Race**				0.525		0.316
Black	383	54.95	152		48	
Mixed race	299	42.90	106		28	
Other	15		4		1	
**Residence**				<0.001		0.591
Urban	573	82.21	234		65	
Rural	124	17.79	28		12	
**TB Bacteriology**	500	27.40	100	<0.001	28	0.245
**TB Medicine Provided**	334	47.92	120	0.385	29	0.056
**TB Notification**	101	14.49	40	0.651	8	0.278
**HIV co-infection**	218	31.28	95	0.053	35	0.005
**Arrived PHC**	262	37.59				
Completed TB Rx	119	17.07				
Cured	65	9.33				
Died	26	3.73				
Defaulted	23	3.30				
Transferred out	11	1.58				
Treatment failure	6	0.86				
**Total Died**	77	11.05				

Multiple logistic regression models found that adults (p < 0.001), urban residence (p < 0.001), TB bacteriology tests (p<0.001), and receiving TB medication (p = 0.021 and p = 0.037 respectively) were predictors of continuity of care for TB patients (including and excluding hospital deaths). An interaction term for age and bacteriology tests for TB was also significant (p = 0.02) for both groups, indicating effect modification between age, TB bacteriology (i.e. children had less bacteriology) and the outcome. Not receiving TB medication was a significant predictor of death for discharged patients (p = 0.032). Table 3.

**Table 3:** Multiple logistic regression of continuity of care for TB patients comparing all TB patients (N = 788) and discharged TB patients who were alive 14 days post discharge (N = 697).

Explanatory Variable	Arrived at another level of care to continue TB treatment						Died after 14 days post discharge		
	All TB patients (N = 788)			TB patients alive 14 days post discharge (N = 697)			TB patients alive 14 days post discharge (N = 697)		
	OR	P value	95% CI	OR	P value	95% CI	OR	P value	95% CI
**Age**	7.14	<0.001	3.04–16.79	7.64	<0.001	3.23–18.03	1.93	0.168	.76–4.91
**Residence**	0.36	<0.001	0.22–0.58	0.35	<0.001	0.21–0.58	0.87	0.683	0.44–1.70
**TB bacteriology**	11.59	<0.001	4.25–31.62	10.63	<0.001	3.87–29.18	1.40	0.610	0.38–5.16
**TB medicine provided**	0.678	0.021	0.49–0.94	0.69	0.037	0.48–0.98	0.57	0.032	0.34–0.95
**HIV co-infection**	0.96	0.037	0.93–0.99	0.96	0.091	0.92–1.01	0.99	0.795	0.94–1.05
**Interaction (Age & TB bact)**	0.28	0.02	0.1–0.82	0.28	0.02	0.10–0.82	1.16	0.83	0.29–4.59
**Cons**	0.09	<0.001	0.04–0.22	0.12	<0.001	.05–0.27	0.08	<0.001	0.03–0.21

## Discussion

Hospitalised TB patients were mostly young, as in previous studies [10, 17], urban and unemployed. The dominance of women in the urban TB population reflects the higher HIV prevalence in female urban TB patient [18]. Re-admissions of a third of TB patients during the six months suggests complex disease and poor quality care [19], and illustrates the dynamic relationship between levels of care.

More CAH patients (74%) had TB bacteriology tests, but a similar proportion received TB treatment (40.7%) to a Kwa-Zulu Natal study (50% tested and 42% treated) [11]. The higher TB bacteriology testing at CAH did not translate into more patients receiving TB treatment. This study however preceded the introduction of the rapid onsite Gene-Expert TB test in South Africa, which was expected to increase TB testing and TB treatment initiation in hospitals and PHC settings[20]. Only 45.3% hospital TB patients had TB drug sensitivity tests, with TB drug resistance levels similar to the national MDR prevalence of 3.5% in new TB patients, and 7.1% in retreatment patients[1].

HIV testing of hospitalised TB patients (24.9%) was lower than the 76% in Gauteng [10] and especially low at the peak of the HIV epidemic [18]. In Africa TB is a leading cause of hospitalisation of people living with HIV, ranging from 8.1% to 41%, and is the cause of 29.3% of deaths in hospitalised adults with HIV [21]. Poor integration of TB and HIV care for co-infected hospital patients contributed to the increased in hospital mortality [21]. The low HIV testing and high mortality in our study suggests more attention needs to be paid to integrating TB and HIV care in hospitals.

The proportion of patients who received TB treatment was based on hospital prescriptions and did not include known TB patients admitted with medication. Treatment practises however did not comply with local policy to provide all TB patients with at least seven days of treatment on discharge from hospital [22].

TB notifications of 13% confirmed previous CAH findings [17], and were lower than the 69% reported elsewhere [10]. This contributes to the large gap between actual incidence of TB and notifications of cases in South Africa [1]. Countries with electronic notification systems have increased TB reporting [23], while South Africa continues to use a paper based notification system not linked to the ETR.net, requiring duplication of reporting by clinicians.

One third of discharged TB patients continued treatment, and one quarter successfully completed treatment, reflecting a failure of TB care pathways. Demographic factors associated with poor continuity of care included children and rural residence. Both groups had less bacteriological confirmation, and more were initiated on treatment without bacteriological confirmation. Hospitals tend to follow up children in outpatient care rather than discharging them to community care, which may have contributed to fewer children in the TB registers [24].

Appropriate clinical management, particularly TB bacteriology testing was strongly associated with better continuity of care. Lack of TB medication on discharge was a predictor of poor continuity of care and patient death in multiple logistic regression models. Culture confirmed paediatric patients < 13 years at CAH had better continuity of care in another study, highlighting the importance of a bacteriological TB diagnosis for continuity of care [24].

Shortcomings in health information systems included the absence of a reliable, integrated hospital TB surveillance system, limiting planning and management of inpatient TB care, hospital infection control, and continuity of care. The hospital and PHC information systems were not linked, impeding follow up of patients and communication between levels of care. We also confirmed findings of low TB notification rates [17], gaps between the ETR.net and facility based TB records in the Western Cape [25], and underreporting of hospital TB patient deaths in the ETR.net [10].

Low numbers of hospital discharged TB patients arriving at PHC services or successfully completing TB treatment reflects poor quality of care, and inefficient linkages between levels of care. The high cost of treating a TB patient in the CAH hospital, $2373 for an average 10 day LOS compared to between $823 and $1362 for community based care per patient cured, further emphasises the need to ensure these scarce resources are used efficiently and effectively[26, 27].

### Strengths and limitations

This study used several sources of routine data, none of which was complete, and extensive data verification was required. Only patients with proof of TB were included, and routine hospital data was verified against patient folders. In the data linkage between hospital data and TB registers, we matched records closely in the electronic systems, and hand searched electronic and paper based TB registers for missing patients.

Only three patients had residential addresses outside the Western Cape, despite evidence that many patients seeking healthcare in the Western Cape originate from neighbouring provinces [28]. The impact of migration for treatment on continuity of TB care could not be assessed in this study.

### Implications for practise and research

Some groups had an increased risk, but all TB patients experienced poor continuity of care. Health systems interventions are needed to improve the quality of hospital TB care, integrate TB and HIV care, establish integrated hospital TB surveillance systems and strengthen referral links between levels of care. The association of TB testing and medication with better continuity of care and patient outcomes warrants further intervention research.

## Conclusion

Most discharged TB patients did not continue care or successfully complete TB treatment with potentially serious implications for patients, the community, and health system. South African hospitals should improve the quality of care of TB patients, develop integrated hospital TB surveillance systems linking to the national TB information system, and strengthen care pathways for TB patients across levels of care.
